# Non-operative management of blunt hepatic and splenic injury: a time-trend and outcome analysis over a period of 17 years

**DOI:** 10.1186/s13017-019-0249-y

**Published:** 2019-06-17

**Authors:** Margot Fodor, Florian Primavesi, Dagmar Morell-Hofert, Veronika Kranebitter, Anna Palaver, Eva Braunwarth, Matthias Haselbacher, Ulrich Nitsche, Stefan Schmid, Michael Blauth, Eva Gassner, Dietmar Öfner, Stefan Stättner

**Affiliations:** 1 Department of Visceral, Transplantation and Thoracic Surgery, Center of Operative Medicine, Medical University of Innsbruck, Anichstraße 35, 6020 Innsbruck, Austria; 2 Department of Radiology, Medical University of Innsbruck, Innsbruck, Austria; 3 Department of Trauma Surgery, Center of Operative Medicine, Medical University of Innsbruck, Innsbruck, Austria; 4 Department of Surgery, Klinikum Rechts der Isar, Technical University of Munich, Munich, Germany; 5 Department of General and Surgical Intensive Care Medicine, Medical University of Innsbruck, Innsbruck, Austria

**Keywords:** Blunt abdominal trauma, Non-operative management, Safety measures

## Abstract

**Background:**

A widespread shift to non-operative management (NOM) for blunt hepatic and splenic injuries has been observed in most centers worldwide. Furthermore, many countries introduced safety measures to systematically reduce severe traffic and leisure sports injuries. This study aims to evaluate the effect of these nationwide implementations on individual patient characteristics and outcomes through a time-trend analysis over 17 years in an Austrian high-volume trauma center.

**Methods:**

A retrospective review of all emergency trauma patients admitted to the Medical University of Innsbruck from 2000 to 2016. Injury severity, clinical data on admission, operative and non-operative treatment parameters, complications, and in-hospital mortality were evaluated.

**Results:**

In total, 731 patients were treated with blunt hepatic and/or splenic injuries. Among these, 368 had a liver injury, 280 splenic injury, and 83 combined hepatic/splenic injury. Initial NOM was performed in 82.6% of all patients (93.5% in hepatic and 71.8% in splenic injuries) with a success rate of 96.7%. The secondary failure rate of NOM was 3.3% and remained consistent over 17 years (*p* = 0.515). In terms of injury severity, we observed a reduction over time, resulting in an overall mortality rate of 4.8% and 3.5% in the NOM group (decreasing from 7.5 to 1.9% and from 5.6 to 1.3%, respectively). These outcomes confirmed an improved utilization of the NOM approach.

**Conclusion:**

Our cohort represents one of the largest Central European single-center experiences available in the literature. NOM is the standard of care for blunt hepatic and splenic injuries and successful in > 96% of all patients. This rate was quite constant over 17 years (*p* = 0.515). Overall, national and regional safety measures resulted in a significantly decreased severity of observed injury patterns and deaths due to blunt hepatic or splenic trauma. Although surgery is nowadays only applied in about one third of splenic injury patients in our center, these numbers might further decrease by intensified application of interventional radiology and modern coagulation management.

**Electronic supplementary material:**

The online version of this article (10.1186/s13017-019-0249-y) contains supplementary material, which is available to authorized users.

## Background

Compared to penetrating injuries, blunt abdominal trauma is very common in central European emergency departments. The management of these injuries can be complex because of frequent association with a multifaceted picture of trauma, involving head, thoracic, and limb injuries [1]. Abdominal organs are involved in approximately 30% of polytrauma patients, with the occurrence of hepatic and splenic injuries in 13 and 16%, respectively [2]. Many technical advances in medicine allow to better diagnose and treat these kinds of injuries both with surgery or non-operative management (NOM), the latter usually also including radiological interventions [3–5].

Currently, NOM is the standard of care in hemodynamically stable patients [6], which is associated with an estimated success rate exceeding 80–90% [2]. NOM has been described as a safe procedure when availability of experienced surgeons, modern imaging modalities, intensive care units (ICU), and other supporting services is assured [7]. While NOM carries the risk of missed hollow visceral injuries or delayed bleeding, operative management (OM) is naturally associated with the possible side effects of any surgical intervention, depending on a variety of patient, medical, and technical factors. In practice, the choice between NOM or OM is mainly driven by hemodynamic considerations rather than the severity of organ injury [8–12]. NOM enables reduction of non-therapeutic laparotomies with potential intra-abdominal complications and unnecessary transfusion risks, thereby resulting in overall lower costs and decreased morbidity and mortality compared to OM [2, 11, 12]. While the benefits of NOM are unquestionable, there is still a lack of consensus regarding patient stratification and potential risk factors for inapplicability or failure of NOM. Strong evidence exists that an age of 40 years or above, Injury Severity Score (ISS) of 25 or greater, associated intra-abdominal injuries and hepatic/splenic injury grade III or higher are prognostic factors for failure of NOM [6, 7, 9, 13].

Regarding the burden of severe injuries, within the last decades, many governments, insurance companies, and automobile clubs in Austria and other European countries introduced a number of measures to improve traffic and sports safety. Examples for preventive traffic measures include nationwide driving license obligations, penalization systems for driving under influence of alcohol and other substances, systematic control of speeding, and compulsory use of helmets for motorcycle drivers and car seat belts especially for children. Concurrent increasing technical standards in the car industry including the development of airbags and driver assistance systems markedly improved safety for traffic participants. Also, across many different types of leisure sports, major efforts were taken, e.g., by introduction of head protection gear in aerial, cycling, and winter sports activities [14], to increase safety.

**Fig. 1:**
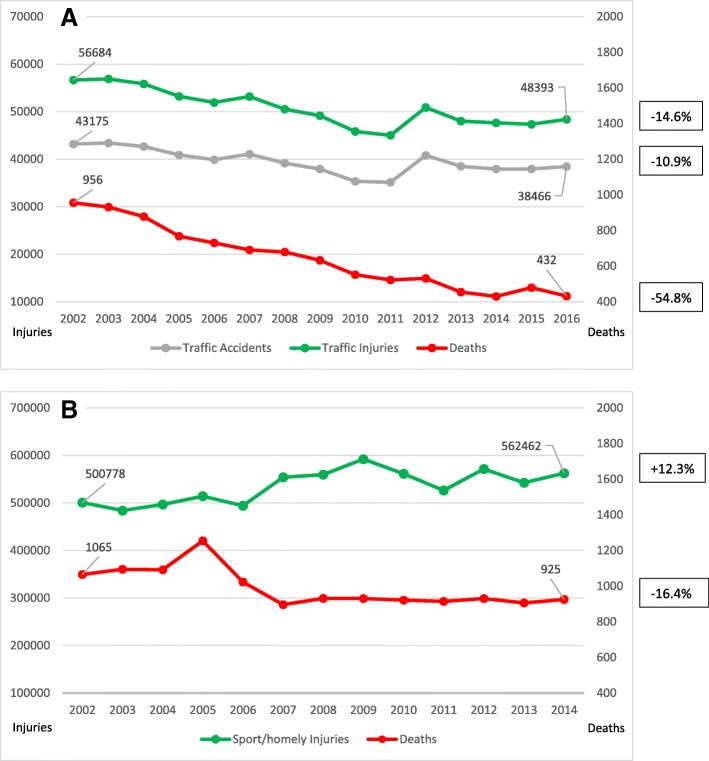
**a** National numbers of road traffic accidents (gray), injured people (green), and deaths (red) in Austria between 2002 and 2016 according to Statistics Austria National Data [14]. **b** Estimated national recreational and sports injuries in Austria from 2002 to 2014 (green) according to the European Injury Database [16]. Road accidents, paid-work accidents, assaults, intentional self-harm, poisoning, and thermal injuries (burn/scald) are excluded. Red: deaths after recreational or sports injuries in Austria according to Statistics Austria National Data [14]

Overall, in the European Union (EU), the number of fatal road crashes decreased by 43% between 2000 and 2010, by another 20% from 2011 to 2017. In this regard, the EU is the world’s safest region with 49 deaths per million inhabitants due to road collisions. However, the safety conditions differ widely between individual member states [15]. In line with the general European trend, also in Austria, fewer road collisions were registered in the last decade. As shown in Fig. 1a, while the number of traffic collisions from 2002 to 2016 in our country decreased by only 10.9% and the number of injured people by 14.6%, traffic deaths could substantially be reduced by 54.8%. The current rate of road deaths/injuries of 0.88% represents the lowest level since the initiation of statistical recording in 1961. In contrast, concerning recreational, sports and homely environment injuries, the number of injured people could not be decreased over the last years in our country (Fig. 1b). Although the death rate is slowly declining also in these types of accidents, some activities such as winter sports are associated with a certain injury risk [14, 16, 17]. Compared to about 8 million skiers and snowboarders visiting the Austrian Alps annually, the mean injury rate is low, at less than 2 injuries per 1000 skier days [17]. However, in 2016, a total of 52.100 winter sports injuries including 40 deaths were registered [14]. Accordingly, national safety bodies have recently stated again that nationwide safety measures have been successfully implemented first and foremost in traffic environment while a comparable effort could not be achieved in another type of injuries so far [18]. Overall, the influence of these national actions on the type and severity of trauma in individual patients and outcomes in specialized centers remains indeterminate.

The objective of this study therefore was to examine the prevalence, clinical presentation, management, and outcomes of OM and NOM of blunt hepatic and splenic injuries over a time span of 17 years at a large tertiary referral center situated in a characteristic central European alpine area in Western Austria.

## Methods

All patients presenting with traumatic blunt hepatic and/or splenic injury to Medical University of Innsbruck from January 1, 2000, to December 31, 2016, were included. Our prospectively maintained and auditable primary patient’s database was retrospectively screened for detailed data about blunt hepatic/splenic injuries from the patients’ medical records and imaging files. To record for consecutive changes in the management of blunt hepatic/splenic trauma (details of patient characteristics, surgical and conservative management, and short-term patient outcome) two comparable distributed subperiods were compared (2000–2008, 2009–2016). Patients who sustained penetrating abdominal injuries and those who were declared dead during transfer to our hospital and were not receiving cardiopulmonary resuscitation (CPR) on arrival were excluded. Patients that were actively treated with fluid or mechanical resuscitation when arriving at our center were included even when no return of spontaneous circulation (ROSC) occurred during further emergency room treatment. Previous external surgical or interventional procedures prior to initial radiographic assessment at our institution were also considered an exclusion criterion, while patients immediately transferred to our center from district hospitals after receiving only diagnostics were included in the analysis. A standardized emergency care protocol is used to manage trauma cases at our center. Focused Assessment with Sonography for Trauma (FAST) is utilized in the early assessment of all patients to detect the presence or absence of hemoperitoneum. Depending on initial assessment, suspected concomitant injuries and X-ray as well as sonography findings, selective abdominal/thoracic/cranial or whole body dual or triple phase CT scan is performed in all hemodynamically stable patients according to a designated protocol. In patients with hemodynamic instability or cases with contraindication for contrast due to severe iodine allergy or poor renal function, sonographic or non-contrast-enhanced CT-assessment was chosen as first assessment. Initial treatment in all patients includes thrombelastography-guided coagulation management according to national guidelines [19], primarily based on individual coagulation factor and fibrinogen substitution, avoidance of fresh frozen plasma, and thrombocyte transfusion when possible and early detection and treatment of deleterious hyperfibrinolysis as previously described by our group [20]. Fluid resuscitation is performed with a balanced combination of crystalloid and colloidal infusions. The trigger level for transfusion of red blood cell and thrombocyte packs has been continuously adapted over the years, currently ranging at a cutoff of 8 g/dl hemoglobin and 100,000/μl thrombocytes, respectively, in our center.

For this study, all CT images were re-evaluated regarding injury grading by two trained radiologists, with a combined clinical experience of > 30 years, blinded to the initial CT result and the clinical outcome. For each patient, 3.5 to 5 mm-thick transversal and 5 mm-thick sagittal and coronal multiplanar-reformatted images were reviewed on a picture archiving and communication system (AGFA IMPAX; AGFA Health Care, Greenville, SC). Divergent findings were jointly assessed, and final results being decided by consensus. Cases in which no lesion was found on radiologic evaluation, with non-existing trauma history (e.g., spontaneous splenic rupture) or with substantially missing information in medical records, were not included in the analysis. For patients with sole sonographic imaging, CT-equivalent injury grading was recorded (all had minor injuries). Blunt hepatic and splenic lesions were classified by the 1994 revision of the AAST-Moore-Classification system (Additional file 1: Table S1 and Additional file 2: Table S2) [21].

The following clinical parameters were collected for this study: patient age and gender, trauma cause, accompanying injuries type and severity, Glasgow Coma Score (GCS), routine laboratory studies, and initial vital parameters. Furthermore, initial trauma management (operative vs. non-operative), type of (N)OM, management complications, failure rate of NOM and underlying failure cause, treatment of complications, total length of hospital stay (LOS), in-hospital mortality, and cause of death were documented. Failure of NOM was defined as the need for operation due to ongoing bleeding from the liver/spleen or other indications for surgery (e.g., abscess, peritonism). ERCP or angiographic interventions were considered as NOM. For each patient, individual injuries were retrospectively classified by body region and by relative severity on a 6-point-scale, according to the latest update (2008) [22] of the Abbreviated Injury Score (AIS) 2005 by the American Association for Automotive Medicine (AAAM) [23]. Injury Severity Scores (ISS) were then calculated upon adding AIS for multiple injured patients [24]. Variables necessary for the Revised Trauma Injury Score (RTS) on admission were not retrospectively available in about half of the patients; thus, this classification was not included in the analysis. The study protocol was approved by the institutional medical review board (protocol-number EK 1034/2017), which waived the need for informed consent due to the retrospective design. The reporting of this study conforms to the STROBE guidelines [25].

### Statistical analysis

Data were presented as proportions (%), means ± standard deviation (SD), or medians as appropriate. Differences between time-periods were determined using the *χ*^2^ and Fisher’s Exact Test for categorical variables and the *T* test or Mann-Whitney *U* test for continuous variables depending on the normal distribution, which was assessed with Shapiro-Wilk test. Two-tailed *p* values less than 0.05 were considered significant throughout the analysis. Data analysis was carried out using SPSS 21.0 (IBM Corporation, Armonk, NY, USA).

## Results

### Patient characteristics

**Fig. 2:**
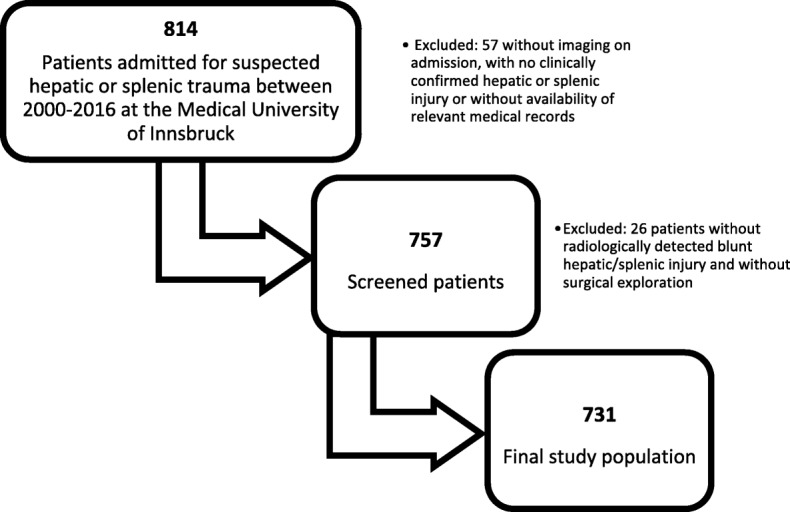
Patient selection chart

Between 2000 and 2016, 814 patients were admitted to our hospital with suspected blunt hepatic or splenic injury (Fig. 2). After exclusion of cases without a history of trauma or without clinical, intraoperative, or radiological confirmation of liver or spleen involvement, a total of 731 patients were included in the final analysis, resulting in a mean of 43 cases per year. Table 1 shows patient characteristics in the whole study period and the differences over time between the early group (2000–2008) and late group (2009–2016). The number of cases with blunt abdominal injuries to the liver and/or spleen was comparable in both periods (*n* = 372 versus *n* = 359), as was in demographic details. However, clinical factors and laboratory findings associated with severity of trauma were markedly different. Patients injured before 2009 showed a significantly lower mean GCS score and higher ISS score (both *p* < 0.001). Concurrently, the rate of patients unconscious on arrival (GCS ≤ 8) decreased significantly (20.7 to 8.6%), as did cases with polytrauma (defined as ISS > 15, 90.9 to 79.1%, both *p* < 0.001). The mean ISS values were lower during the recent period in both operatively and non-operatively managed patients (*p* < 0.001).

**Table 1:** Patient characteristics and admission values

	Overall cohort (2000–2016) *n* = 731	Early period (2000–2008) *n* = 372	Late period (2009–2016) *n* = 359	Difference between periods *P*
Male	497 (68%)	251 (67.5%)	246 (68.5%)	0.761
Age (mean, SD)	32.4 (18.1)	31.8 (17.8)	33.1 (18.4)	0.318
GCS on admission *(missing = 15)*				
Mean (SD)	13.02 (3.57)	12.46 (3.9)	13.60 (3.1)	< 0.001
GCS ≤ 8	106 (14.8%)	76 (20.7%)	30 (8.6%)	< 0.001
Injury Severity Score				
Median (range)	27 (4–75)	33 (4–75)	24 (4–65)	< 0.001
> 15 (definition of polytrauma)	622 (85.1%)	338 (90.9%)	284 (79.1%)	< 0.001
Mean (SD) NOM patients	26.7 (12.35)	29.3 (12.24)	24.1 (11.92)	< 0.001
Mean (SD) OM patients	31.5 (13.86)	35.8 (12.39)	26.3 (13.84)	< 0.001

*GCS* Glasgow Coma Scale, *SD* standard deviation, *NOM* non-operative management, *OM* operative management

### Injury details

Details of trauma characteristics, involving organs and severity of liver and spleen injury according to AAST classification are depicted in Table 2. Concerning the mechanisms of injury, also significant changes over the two periods could be shown (*p* < 0.001). While previously traffic incidents involving a car, motorcycle, and pedestrian collision were accounting for 46.7% of injuries, these declined to 29.2% in recent years. Simultaneously, winter sport and cycling collision were increasing from 31.4 to 52.4%. Other causes of trauma included fall from heights, horse riding collision, minimal trauma (mostly in homely environment), and exceptionally rarely personal assault. As a result, in terms of involved organs, combined liver and spleen injury decreased significantly from 15.3 to 7.2%, with a concurrent rise in isolated splenic trauma (33.1 to 43.7%, *p* = 0.001). Overall, the cohort included 280 splenic, 368 liver, and 83 combined liver and spleen injuries.

**Table 2:** Trauma characteristics

	Overall cohort (2000–2016) *n* = 731	Early period (2000–2008) *n* = 372	Late period (2009–2016) *n* = 359	Difference between periods *P*
a. Trauma mechanism injury data				
Trauma cause (missing = 2)				
Car collision	156 (21.4%)	101 (27.3%)	55 (15.3%)	< 0.001
Motorcycle collision	87 (11.9%)	46 (12.4%)	41 (11.4%)	
Pedestrian or comparable occupational collision	35 (4.8%)	26 (7%)	9 (2.5%)	
Cycling collision	62 (8.5%)	24 (6.5%)	38 (10.6%)	
Winter sports	242 (33.2%)	92 (24.9%)	150 (41.8%)	
Fall from heights	76 (10.4%)	49 (13.2%)	27 (7.5%)	
Trauma in homely environment	47 (6.4%)	20 (5.4%)	27 (7.5%)	
Personal assault	3 (0.4%)	1 (0.3%)	2 (0.6%)	
Horse riding collision (and other animal associated injuries)	21 (2.9%)	11 (3.0%)	10 (2.8%)	
b. Organ injury data				
Injured organ				
Spleen	280 (38.3%)	123 (33.1%)	157 (43.7%)	0.001
Liver	368 (50.3%)	192 (51.6%)	176 (49%)	
Combined liver and spleen injury	83 (11.4%)	57 (15.3%)	26 (7.2%)	
AAST (Moore) Injury Score (according to admission CT scan; no CT, *n* = 9)				
Spleen				
No spleen injury	367 (50.8%)	192 (52.7%)	175 (48.9%)	0.008
I	40 (5.5%)	22 (6%)	18 (5%)	
II	66 (9.1%)	40 (11%)	26 (7.3%)	
III	173 (24.0%)	76 (20.9%)	97 (27.1%)	
IV	47 (6.5%)	15 (4.1%)	32 (8.9%)	
V	29 (4.0%)	19 (5.2%)	10 (2.8%)	
Liver				
No liver injury	277 (38.4%)	120 (33.0%)	157 (43.9%)	0.096
I	43 (6%)	21 (5.8%)	22 (6.1%)	
II	97 (13.4%)	52 (14.3%)	45 (12.6%)	
III	221 (30.6%)	124 (34.1%)	97 (27.1%)	
IV	66 (9.1%)	37 (10.2%)	29 (8.1%)	
V	17 (2.4%)	9 (2.5%)	8 (2.2%)	
VI	1 (0.1%)	1 (0.3%)	0 (0%)	
Associated extra-abdominal injuries (AIS ≥ 1)				
Head or neck	267 (36.5%)	173 (46.5%)	94 (26.2%)	< 0.001
Face	101 (13.8%)	52 (14%)	49 (13.6%)	0.897
Chest	463 (63.3%)	270 (72.6%)	193 (53.8%)	< 0.001
Extremities or pelvic girdle	342 (46.8%)	203 (54.6%)	139 (38.7%)	< 0.001
External (skin and soft tissue)	457 (62.5%)	291 (78.2%)	166 (46.2%)	< 0.001

*ISS* Injury Severity Score, *AAST* American Association for the Surgery of Trauma, *AIS* Abbreviated Injury Score

Regarding grading according to the AAST classification, in the early period, 54.2% of patients had minor liver trauma (AAST I-III) and 37.9% minor spleen trauma versus 45.8% and 39.4% in the late period, respectively.

Concerning extra-abdominal injuries, all body regions except face were significantly less often involved over the analyzed time span (each *p* < 0.001).

### Management of trauma patients (Table 3)

One hundred twenty-seven (17.4%) patients underwent immediate surgery, with no significant change over time (*p* = 0.393). Complete splenectomy was performed in 79 (10.8%) patients, while spleen-preserving procedures were applied in 17 (2.3%) cases. Liver resection was performed in 8 (1.1%) patients, liver-conserving procedures were sufficient in 34 (4.7%) patients (suture *n* = 16, hemostasis *n* = 10, packing *n* = 8 patients).

**Table 3:** Management of blunt hepatic and splenic injuries

	Overall cohort (2000–2016) *n* = 731	Early period (2000–2008) *n* = 372	Late period (2009–2016) *n* = 359	Difference between periods *P*
Primary therapy				
OM	127 (17.4%)	69 (18.5%)	58 (16.2%)	0.393
NOM	604 (82.6%)	303 (81.5%)	301 (83.8%)	
NOM rate according to the injured organ				
NOM splenic injury	201 (71.8%)	85 (69.1%)	116 (73.9%)	0.378
NOM hepatic injury	344 (93.5%)	177 (92.2%)	167 (94.9%)	0.295
NOM combined splenic and hepatic injury	59 (71.1%)	41 (71.9%)	18 (69.2%)	0.801
Surgical procedures (% of all patients)				
Spleen				
Complete splenectomy	79 (10.8%)	41 (11%)	38 (10.6%)	0.966
Spleen-conserving procedures (suture, hemostasis, etc.)	17 (2.3%)	9 (2.4%)	8 (2.2%)	
Liver				
Hepatic partial resection	8 (1.1%)	5 (1.3%)	3 (0.8%)	0.113
Liver-conserving procedure (suture, hemostasis, etc.)	34 (4.7%)	23 (6.2%)	11 (3.1%)	
Angiography or ERCP in case of primary NOM	9 (1.2%)	2 (0.5%)	7 (2.0%)	0.194
Failure NOM (secondary operative, % of NOM)	20 (3.3%)	11 (3.6%)	9 (3%)	0.515
Cause for the failure of NOM (% of all NOM)				
Persistent or secondary bleeding/hemodynamic instability	13 (65%)	5 (45.5%)	8 (88.9%)	0.123
Extent of intra-abdominal hematoma (compression/compartment)	5 (25%)	4 (36.4%)	1 (11.1%)	
Infectious complications (bile duct injury, sepsis, MOF)	2 (10%)	3 (18.2%)	0 (0%)	
Management of failed NOM (% of all failed NOM)				
Splenectomy	11 (55%)	5 (45.5%)	6 (66.7%)	0.517
Spleen-conserving surgery	2 (10%)	2 (18.2%)	0 (0%)	
Liver resection	3 (15%)	1 (9.1%)	2 (22.2%)	
Non-resection liver surgery (suture, hemostatics, etc.)	4 (20%)	3 (27.3%)	1 (11.1%)	

*OM* operative management, *NOM* non-operative management, *ERCP* endoscopic retrograde cholangiopancreatography, *SD* standard deviation, *MOF* multiorgan failure

In contrast, 604 (82.6%) patients underwent initial non-operative treatment, with a total of 344 hepatic (93.5%), 201 splenic (71.8%), and 59 combined injuries (71.1%) being managed non-operatively. Also, no significant differences were noted over time.

In all patients undergoing primary OM, hemodynamic instability was persistent despite appropriate resuscitation measures (as described in the “Methods” section), and instability therefore was the primary indication for surgery. Associated radiological or laboratory findings recorded (multiple possible) were high organ-specific injury score (*n* = 36, 28.4%), involvement of multiple abdominal organs (*n* = 46, 36.2%), extensive hemoperitoneum (*n* = 34, 26.8%), substantial drop in hemoglobin with need for massive transfusion (*n* = 33, 26%), and marked extravasation of contrast media on CT (*n* = 13, 10.2%). In 47 patients (37%), no recordings of associated findings were available in our records.

NOM was successful in 584 patients (96.7% of NOM cases). Twenty failures (3.3%) were observed, and this rate was consistent over 17 years (*p* = 0.515). Underlying causes for NOM failure did change over time with the most relevant cause being hemodynamic instability due to persistent or secondary bleeding lately (90% compared to previously 45.5%); however, this was not statistically significant (*p* = 0.123). The management for failure of NOM patients included splenectomy (55%), spleen-conserving surgery (10%), liver resection (15%), and non-resecting liver surgery (20%) including suture (*n* = 1) and hemostatics (*n* = 3).

### Outcome of trauma patients (Table 4)

Median duration of hospital stay was 14 days (SD 20.2) with a range of 0–382 days, significantly decreasing from 16 to 13 days in the recent period (*p* < 0.001).

**Table 4:** Outcomes of treatment for blunt hepatic and splenic injuries

Length of hospital stay (days, missing = 4)				
Median (range)	14 (0–382)	16 (0–382)	13 (0–112)	< 0.001
In-hospital mortality (% of all patients)	35 (4.8%)	28 (7.5%)	7 (1.9%)	< 0.001
Mortality of primary NOM patients	21 (3.5%)	17 (5.6%)	4 (1.3%)	0.006
Mortality of primary surgical patients	14 (11%)	11 (15.9%)	3 (5.2%)	0.086
Cause of death (% of deaths)				
Sepsis	7 (20%)	7 (25%)	0 (0%)	0.436
Hemorrhagic shock	3 (8.6%)	3 (10.7%)	0 (0%)	
Intracranial hypertension	11 (31.4%)	8 (28.6%)	3 (42.9%)	
Multiorgan failure	5 (14.3%)	3 (10.7%)	2 (28.6%)	
Cardiac dysfunction/infarction	1 (2.9%)	1 (3.6%)	0 (0%)	
Arrived with CPR, no ROSC	6 (17.1%)	5 (17.9%)	1 (14.3%)	
Other/unknown	2 (5.7%)	1 (3.6%)	1 (14.3%)	

*CPR* cardiopulmonary resuscitation, *ROSC* return of spontaneous circulation

Overall mortality in the study cohort was 4.8%, with 11% in the surgery group and 3.5% in the NOM group. Mortality in failure of NOM patients was 0% compared to 3.6% (*n* = 21) in patients without failure of NOM. Over time, a significant decrease in overall mortality (7.5 to 1.9%; *p* < 0.001) and mortality in the conservatively managed subgroup (from 5.6 to 1.3%, *p* = 0.006) was confirmed by our analysis. While an even more pronounced improvement in mortality was noted in the operative group between the two study periods (15.9 to 5.2%), this did just not reach statistical significance (*p* = 0.086), primarily owed to the limited number of OM patients in both groups.

Most frequent cause of death was uncontrollable intracranial hypertension in 11 (31.4%) patients, followed by septic shock (*n* = 7, 20%), and no return of spontaneous circulation (ROSC) after cardiopulmonary resuscitation (CPR) on arrival.

To compare outcomes of blunt hepatic or splenic injury patients with our overall emergency care outcome, we further assessed the number of total injury-related admissions to our emergency room and the respective death rate over time (available from 2007 to 2016). During three equal periods, the mortality decreased from 7.6% (*n* = 136/1791) in 2007–2010 to 6.8% (*n* = 125/1843) in 2011–2013 and 6.3% (109/1724) in the recent time-period (2014–2016); however, with no statistical significance (*p* = 0.321). While admission rates remain constant, this accounts for an absolute decrease in mortality of 1.3% over 10 years (17% relative risk reduction).

### Differences over time between operative and non-operative managed patients

Changes in patient characteristics and management details of OM and NOM over the two periods are summarized in Table 5. Regarding ISS comparing OM and NOM patients, a significant difference was observed in the early period (*p* = 0.046), while the severity of the injury was identical between NOM and OM cases recently (*p* = 0.507). Also, relevant differences in the management of blunt injuries were registered over both periods: OM was mostly applied in isolated splenic trauma, while isolated liver injuries were primarily treated with NOM (*p* < 0.001) in both periods. Significant differences concerning performed therapy according to the severity of organ injury were detected: in both periods, high-grade splenic injuries were mostly treated with operative therapy. In patients with liver trauma however, the rate of high-grade injuries requiring operative management decreased over time from 19.5 to 10.5%. We furthermore analyzed the differences in the success of NOM between patients with low-grade (AAST I–III) versus high-grade (≥ IV) splenic and/or liver injuries over time. In the whole observation period, failure of NOM occurred in 2% of low-grade injuries compared to 10.4% in high-grade injuries (*p* < 0.001). Interestingly, the rate was 1.9% versus 13.3% in the early period (*p* < 0.001) compared to 2% versus 7.8% (*p* = 0.048) in the late period.

**Table 5:** Differences between operative (OM) and non-operative managed (NOM) patients in the two periods

	Early period (2000–2008)			Late period (2009–2016)		
	OM patients *n* = 69	NOM patients *n* = 303	*P*	OM patients *n* = 58	NOM patients *n* = 301	*P*
Male	52 (75.4%)	199 (65.7%)	0.121	40 (69.0%)	206 (68.4%)	0.937
Age (median, SD)	31.8 (17.2)	26.5 (17.9)	0.087	34.5 (17.9)	29.0 (18.5)	0.355
Trauma cause (missing = 2)						
Car collision	16 (23.2%)	85 (28.2%)	0.518	9 (15.5%)	46 (15.3%)	0.322
Motorcycle collision	15 (21.7%)	31 (10.3%)		6 (10.3%)	35 (11.6%)	
Pedestrian or occupational collision	4 (5.8%)	22 (7.3%)		2 (3.4%)	7 (2.3%)	
Cycling collision	5 (7.2%)	19 (6.3%)		2 (3.4%)	36 (12.0%)	
Winter sports	15 (21.7%)	77 (25.6%)		26 (44.8%)	124 (41.2%)	
Fall from heights	8 (11.6%)	41 (13.6%)		6 (10.3%)	21 (7.0%)	
Trauma in homely environment	4 (5.8%)	16 (5.3%)		7 (12.1%)	20 (6.6%)	
Personal assault	0 (0%)	1 (0.3%)		0 (0%)	2 (0.7%)	
Horse riding collision (and other animals)	2 (2.9%)	9 (3.0%)		0 (0%)	10 (3.3%)	
Glasgow Coma Scale (GCS) on admission ≤ 8	20 (29%)	56 (18.7%)	0.058	5 (8.9%)	25 (8.5%)	0.923
Injury Severity Score (ISS) > 15	67 (97.1%)	271 (89.4%)	0.046	44 (75.9%)	240 (79.7%)	0.507
Injured organ						
Spleen	38 (55.1%)	85 (28.1%)	< 0.001	41 (70.7%)	116 (38.5%)	< 0.001
Liver	15 (21.7%)	177 (58.4%)		9 (15.5%)	167 (55.5%)	
Combined liver and spleen injury	16 (23.2%)	41 (13.5%)		8 (13.8%)	18 (6%)	
AAST (Moore) Injury Score (missing = 9)						
Spleen						
No spleen injury	15 (24.2%)	177 (58.6%)	< 0.001	8 (14%)	167 (55.5%)	< 0.001
I–III	23 (37.1%)	115 (38.1%)		27 (47.4%)	114 (37.9%)	
IV–V	24 (38.7%)	10 (3.3%)		22 (38.6%)	20 (6.6%)	
Liver						
No liver injury	36 (58.1%)	84 (27.8%)	< 0.001	41 (71.9%)	116 (38.5%)	< 0.001
I–III	14 (22.6%)	183 (60.6%)		10 (17.5%)	154 (51.2%)	
IV–VI	12 (19.4%)	35 (11.6%)		6 (10.5%)	31 (10.3%)	
Associated extra-abdominal injuries (AIS ≥ 1)						
Head or neck	35 (50.7%)	138 (45.5%)	0.436	12 (20.7%)	82 (27.2%)	0.299
Face	10 (14.5%)	42 (13.9%)	0.891	5 (8.6%)	44 (14.6%)	0.223
Chest	49 (71%)	221 (72.9%)	0.747	35 (60.3%)	158 (52.5%)	0.272
Extremities or pelvic girdle	44 (63.8%)	159 (52.5%)	0.089	22 (37.9%)	117 (38.9%)	0.893
External (skin and soft tissue)	57 (82.6%)	234 (77.2%)	0.328	30 (51.7%)	136 (45.2%	0.360

*OM* operative management, *NOM* non-operative management, *SD* standard deviation, *ISS* Injury Severity Score, *AAST* American Association for the Surgery of Trauma, *AIS* Abbreviated Injury Score, *GCS* Glasgow Coma Scale

## Discussion

This analysis is characterized by three main points: (I) the observed cohort represents one of the largest Western European single-center experiences available, reporting clinical presentations, management, and outcomes of blunt liver and splenic injuries over a time span of 17 years; (II) NOM was the option of treatment in more than 80% of cases with only minor changes over time; and (III) injury severity decreased over time accompanied by major improvements in mortality rates.

The mechanisms of liver and splenic injuries vary geographically due to community factors [26]. Compared to other continents, blunt abdominal trauma comprises most of the injuries in the EU and typically results from a motor vehicle collision or a fall, while penetrating injuries as a result of bullet or knife assaults account for a minimal amount of trauma. In contrast to other national studies, where the rate of blunt abdominal injuries is high secondary to the increasing trend of motor vehicles [26], in Austria, traffic collisions declined in the last years *(*Fig. 1a). In contrast, a growing number of polytrauma caused by recreational activities were registered over the recent decades *(*Fig. 1b) [17]. From 2002 to 2014, overall injury rate increased about 12.3%, and in contrast, death rate decreased about 16.4% [16]. Previous studies showed that concerning sport activities, most injuries occurred by practicing soccer or winter sports [18].

This was also confirmed by the present analysis were currently winter sports are responsible for more than 40% of splenic or hepatic injuries, by far representing the major trauma mechanism in patients admitted to our center. The persisting large number of severe winter sports accidents in our catchment area might be explained by dramatic increases of tourism accompanied by over-crowded slopes and additional aggravation through the invention of new high-speed sports equipment such as carving skies. Simultaneously, for example, head protection gear is still not mandatory above the age of 15 in our state.

In terms of injury severity, we observed a significant reduction over time through several parameters including ISS, GCS, and organ involvement patterns. Alongside improved medical, surgical, and intensive care trauma treatment in our center, we believe that nationwide safety measures are substantially responsible for this trend resulting in markedly reduced regional and national trauma mortality (Fig. 1a, b) [18].

In our study, the overall mortality rate was 4.8% (11% in the OM group and 3.5% in the NOM group), and most deaths were due to extensive concomitant extra-abdominal injuries, resulting in a subsequent septic shock or unmanageable intracranial hypertension. The mortality rate in the whole cohort as well as in the NOM subgroup decreased significantly over the study period (7.5 to 1.9% and 5.6 to 1.3%, respectively), a similar trend was observed in the operative group (15.9 to 5.2%), although closely not statistically significant (*p* = 0.086). Our results are in line with other published data showing overall mortality rates around 3.5% [27].

According to data from the World Health Organization (WHO), 1.24 million road traffic deaths occur every year, differing widely between individual continents. In the African continent, the highest rate (24.1 per million population) is described, while in South-East Asia and Western Pacific 18.5 deaths per million are counted. The American and European continents are still the safest regions with 16.1 and 10.3 deaths per million, respectively. Simultaneous to the development of the newest medical therapies, the introduction of national safety measures dramatically contributed to a relevant decline of road and sports collisions. As an example, 5% cut in average speed can result in 30% reduction of fatal crashes, as well as wearing a motorcycle helmet correctly ends in 40% reduction of death risk. Finally, seat belts reduce the risk of a fatal injury in 50–75% [28]. The effect of such legislations moderated the injury grade and the involvement of extra-abdominal regions, ameliorating the injury prognosis.

Regarding trauma management, previous studies demonstrated that around 80% of patients with hepatic or splenic injuries can successfully be treated conservatively [2]. Reported failure rates reached from 3 to 10% and were mainly secondarily to delayed bleeding, hematoma, and associated injuries [26]. In line with this, our results showed 96.7% NOM success rate. Failure of NOM amounts to 3.3% over the study period, caused by hemodynamic instability due to persistent or secondary bleeding. These outcomes, in view of a significant decrease in mortality in conservatively treated patients (from 5.6 to 1.3%), indicate an improved utilization of the NOM approach as compared to initial series where 60% of cases were treated conservatively with a subsequent failure rate of 15% [29]. Modern coagulation management, including thromboelastography to guide blood product resuscitation, contributed to improved outcomes for NOM at our center. According to an Austrian survey in 2016, the rate of NOM was > 50% in more than two thirds of all national hospitals and an increasing trend towards radiologic interventions was reported [30].

In the present study, 17.4% of cases underwent OM due to hemodynamic instability. Co-factors included high injury score and multiple abdominal injuries. Notably, OM was primarily applied in high-grade splenic injuries over the whole study period and high-grade liver injuries in the early period, while major hepatic injuries in the late period were treated equally with OM and NOM (10.5% vs 10.3%). With ongoing specialization and more complex procedures in liver surgery showing its unique regeneration potential, more conservative approaches even in higher grade liver injuries were utilized. Moreover, a detailed radiological scoring system (AAST) has given us the ability to characterize injury severity more precisely. However, the AAST-Moore-Classification does not incorporate the full spectrum of vascular injuries and might therefore be of less value. The Baltimore CT severity index (CTSI) for splenic injuries tries to overcome this limitation and allows for direct correlation of vascular CT imaging characteristics and choice of patient clinical management [31]. The purpose and validation of a similar score for liver injuries are currently under development at our institution.

Although most solid organ injuries can be treated with NOM, mainly due to improvement in overall interdisciplinary trauma management, the ones that required surgery in our unit did so emergently, carrying a high risk of morbidity and mortality if appropriate surgical treatment is delayed. In order to further raise the rate of splenic NOM management, increased application of interventional radiology may be a key feature. In contrast with the increasing utilization in other level I trauma centers, in our series, angiography or ERCP were applied in a low number of only 1.2% in case of primary NOM, showing a clear potential for improvement. Overcoming the limited experience of the last years, however, these techniques have gained expertise and acceptance recently at our center. Interventional radiology techniques, especially in case of contrast extravasation, were previously described as important diagnostic and therapeutic tools in a relevant number of patients with blunt liver and splenic injuries to improve NOM success rates and reduce avoidable operations [9, 32, 33]. The organ salvage rate ranges from 86 to 100%, with success rates > 90% in splenic injuries and between 79% and 92% in liver injuries by using arterial embolization [34–36]. Even if morbidity and complication rates like re-bleeding should be considered after angiography [9], further improvements in overall mortality by increased implementation of these techniques and modern coagulation management could be expected in our center [37, 38].

There were several limitations to the current study, mostly due to the retrospective study design. For example, detailed information regarding concomitant injured abdominal organs, as well as operation times, post-operative complications, and follow-up post-discharge were not recorded systematically and would be of interest in further prospective studies. Additionally, the RTS classification was excluded from data analysis because of a large number of missing hemodynamic variables. Furthermore, pre-hospital or on admission death patients were not included in this analysis which may underestimate the regional on-site mortality rate due to blunt trauma. Finally, the strict focus on local patients with hepatic and splenic injuries may limit the ability to extrapolate our results to overall changes of injury rates and trauma outcomes on a broader level. However, by implementing national trauma data as well as overall hospital emergency admission data, we have aimed to further strengthen our conclusions. This analysis represents one of the largest single-center experiences on blunt hepatic and splenic injuries performed over a long study period to be reported in Western Europe. Besides systematically re-evaluated radiological reports, it includes extensive data on injury patterns, surgical, and NOM details as well as outcome parameters and therefore clearly improves available knowledge in this specific subject.

## Conclusion

The present study confirmed the effectiveness of NOM in patients with liver and splenic injuries, suggesting a safe and effective therapeutic approach, with recent overall mortality rates below 2%. According to these findings, the majority of patients can be treated with NOM in a less invasive manner, avoiding unnecessary laparotomies, which is particularly true in liver injuries with recent NOM rates surpassing 95%. Although surgery is nowadays only applied in about one third of splenic injury patients in our center, these numbers might further decrease by intensified application of interventional radiology. Overall, national and regional safety measures resulted in a significantly decreased severity of observed injury patterns and deaths due to blunt hepatic or splenic trauma. Further prospective studies in high-volume centers or on a national level including analysis of pre-hospital management and systematic implementation of interventional radiology would be desirable to validate these findings and further improve patient care.

## Additional files

Additional file 1: **Table S1.** Moore classification/AAST liver injury scale [1]. (DOCX 17 kb)

Additional file 2: **Table S2.** Moore classification/AAST spleen injury scale [1]. (DOCX 17 kb)

## Data Availability

All data generated or analyzed during this study are included in this published article and its supplementary information files.

## Abbreviations

AAAM: American Association for Automotive Medicine
AAST: American Association for Surgery of Trauma
AIS: Abbreviated Injury Score
CPR: Cardiopulmonary Resuscitation
CTSI: CT severity index
ERCP: Endoscopic retrograde cholangiopancreatography
EU: European Union
GCS: Glasgow Coma Score
ISS: Injury Severity Score
LOS: Length of hospital stay
NOM: Non-operative management
OM: Operative management
ROSC: Return of spontaneous circulation
