# Supplementary material for: Non-operative management of blunt hepatic and splenic injury: a time-trend and outcome analysis over a period of 17 years
Source: World J Emerg Surg. 2019 Jun 17;14:29. doi: 10.1186/s13017-019-0249-y (PMC6580509; doi:10.1186/s13017-019-0249-y)
Supplement: Supplementary file 1 — Table S1. Moore classification/AAST liver injury scale [1]. (DOCX 17 kb) [file 13017_2019_249_MOESM1_ESM.docx]

**Additional file 1: Table S1**: Moore classification / AAST liver injury scale (1)

| GRADE | TYPE |  | INJURY DESCRIPTION |
| --- | --- | --- | --- |
| I | Haematoma  Laceration |  | Subcapsular, < 10 % surface area  Capsular tear, < 1 cm parenchymal depth |
| II | Haematoma  Laceration |  | Subcapsular, 10-50 % surface area, intra-parenchymal < 10 cm in diameter  1-3 cm parenchymal depth, < 10 cm in length |
| III | Haematoma  Laceration |  | Subcapsular, > 50 % surface area or expanding; ruptured subcapsular or parenchymal haematoma, intra-parenchymal haematoma ≥ 10 cm or expanding  > 3 cm parenchymal depth |
| IV | Laceration |  | Parenchymal disruption involving 25-75 % of hepatic lobe or 1-3 Couinaud´s segments within the single lobe |
| V | Laceration  Vascular |  | Parenchymal disruption involving > 75 % of hepatic lobe or > 3 Couinaud´s segments within the single lobe  Juxtavenous hepatic injuries; i.e. retrohepatic vena cava/central major hepatic veins |
| VI | Vascular |  | Hepatic avulsion |

*Advance one grade for multiple injuries, up to grade III
