# Supplementary material for: Non-operative management of blunt hepatic and splenic injury: a time-trend and outcome analysis over a period of 17 years
Source: World J Emerg Surg. 2019 Jun 17;14:29. doi: 10.1186/s13017-019-0249-y (PMC6580509; doi:10.1186/s13017-019-0249-y)
Supplement: Supplementary file 2 — Table S2. Moore classification/AAST spleen injury scale [1]. (DOCX 17 kb) [file 13017_2019_249_MOESM2_ESM.docx]

**Additional file 2: Table S2**: Moore classification / AAST spleen injury scale (1)

| GRADE | TYPE |  | INJURY DESCRIPTION |
| --- | --- | --- | --- |
| I | Haematoma  Laceration |  | Subcapsular, < 10 % surface area  Capsular tear, < 1 % parenchymal depth |
| II | Haematoma  Laceration |  | Subcapsular, 10-50 % surface area, intra-parenchymal < 5 cm in diameter  Capsular tear, 1-3 cm parenchymal depth that does not involve a trabecular vessel |
| III | Haematoma  Laceration |  | Subcapsular, > 50 % surface area or expanding; ruptured subcapsular or parenchymal haematoma, intra-parenchymal haematoma ≥ 5 cm or expanding  > 3 cm parenchymal depth or involving trabecular vessels |
| IV | Laceration |  | Laceration involving segmental or hilar vessels producing major devascularisation (> 25 % of spleen) |
| V | Laceration  Vascular |  | Complete shattered spleen  Hilar vascular injury which devascularizes spleen |

*Advance one grade for multiple injuries, up to grade III
